# Sylvatic Plague Vaccine Partially Protects Prairie Dogs (*Cynomys* spp.) in Field Trials

**DOI:** 10.1007/s10393-017-1253-x

**Published:** 2017-06-22

**Authors:** Tonie E. Rocke, Daniel W. Tripp, Robin E. Russell, Rachel C. Abbott, Katherine L.D. Richgels, Marc R. Matchett, Dean E. Biggins, Randall Griebel, Greg Schroeder, Shaun M. Grassel, David R. Pipkin, Jennifer Cordova, Adam Kavalunas, Brian Maxfield, Jesse Boulerice, Michael W. Miller

**Affiliations:** 1 U.S. Geological Survey, National Wildlife Health Center, 6006 Schroeder Rd., Madison, WI 53711 USA; 2 Colorado Division of Parks and Wildlife, Wildlife Health Program, 4330 Laporte Avenue, Fort Collins, CO USA; 3 U.S. Fish and Wildlife Service, Charles M. Russell National Wildlife Refuge, Lewistown, MT USA; 4 U.S. Geological Survey, Fort Collins Science Center, 2150 Centre Ave, #C, Fort Collins, CO USA; 5 U.S. Forest Service, P.O. Box 425, Wall, SD USA; 6 U.S. National Park Service, Wind Cave National Park, 26611 Highway 385, Hot Springs, SD USA; 7 Lower Brule Sioux Tribe, Department of Wildlife, Fish and Recreation, P.O. Box 246, Lower Brule, SD USA; 8 U.S. Department of Agriculture, APHIS, Wildlife Services, WTAMU, P.O. Box 60277, Canyon, TX USA; 9 Arizona Game and Fish Department, P.O. Box 397, Seligman, AZ USA; 10 Utah Division of Wildlife Resources, 1470 North Airport Rd., Cedar City, UT USA; 11 Utah Division of Wildlife Resources, 318 North Vernal Ave., Vernal, UT USA; 12 Wyoming Game and Fish Department, 528 South Adams Street, Laramie, WY USA

**Keywords:** *Cynomys* spp., Sylvatic plague, Prairie dogs, Vaccine, *Yersinia pestis*

## Abstract

Sylvatic plague, caused by *Yersinia pestis*, frequently afflicts prairie dogs (*Cynomys* spp.), causing population declines and local extirpations. We tested the effectiveness of bait-delivered sylvatic plague vaccine (SPV) in prairie dog colonies on 29 paired placebo and treatment plots (1–59 ha in size; average 16.9 ha) in 7 western states from 2013 to 2015. We compared relative abundance (using catch per unit effort (CPUE) as an index) and apparent survival of prairie dogs on 26 of the 29 paired plots, 12 with confirmed or suspected plague (*Y. pestis* positive carcasses or fleas). Even though plague mortality occurred in prairie dogs on vaccine plots, SPV treatment had an overall positive effect on CPUE in all three years, regardless of plague status. Odds of capturing a unique animal were 1.10 (95% confidence interval [C.I.] 1.02–1.19) times higher per trap day on vaccine-treated plots than placebo plots in 2013, 1.47 (95% C.I. 1.41–1.52) times higher in 2014 and 1.19 (95% C.I. 1.13–1.25) times higher in 2015. On pairs where plague occurred, odds of apparent survival were 1.76 (95% Bayesian credible interval [B.C.I.] 1.28–2.43) times higher on vaccine plots than placebo plots for adults and 2.41 (95% B.C.I. 1.72–3.38) times higher for juveniles. Our results provide evidence that consumption of vaccine-laden baits can protect prairie dogs against plague; however, further evaluation and refinement are needed to optimize SPV use as a management tool.

**Electronic supplementary material:**

The online version of this article (doi:10.1007/s10393-017-1253-x) contains supplementary material, which is available to authorized users.

## Introduction

Controlling disease in free-ranging wildlife populations poses formidable challenges. In the last few decades, new techniques have been developed, including oral vaccines that can be delivered en masse to wild animals via baits. For example, rabies has been curtailed in carnivore populations in both Europe and North America by distributing baits that contain recombinant vaccinia virus expressing rabies glycoprotein G (Freuling et al. 2013; Slate et al. 2009). Other vaccines currently being developed for oral delivery to wildlife include chimeric viruses for controlling classical swine fever in wild boar (Rossi et al. 2015) and vaccinia-vectored vaccines against Lyme disease in rodents (Bhattacharya et al. 2011) and tuberculosis in European badgers (*Meles meles*) (Murphy et al. 2014). In these cases, the primary goal of vaccination is to reduce wildlife reservoirs of diseases affecting humans and domestic animals. Recently, a vaccine against sylvatic plague, a flea-borne disease of wild rodents caused by *Yersinia pestis*, was developed for use in prairie dogs (*Cynomys* spp.), primarily for the conservation of threatened and endangered species (Abbott et al. 2012), although lowering risk of *Y. pestis* exposure to humans and domestic animals would be an additional benefit.

Since its introduction to the continent more than 100 years ago, sylvatic plague has resulted in major disruptions in North American ecosystems (Eads and Biggins 2015) and contributed to the decline of several threatened and endangered species, including the Utah prairie dog (*C. parvidens*) and the black-footed ferret (*Mustela nigripes*). Plague is widespread throughout the western USA and frequently occurs in wild rodents. All four species of prairie dogs in the USA are particularly susceptible to plague, suffering high mortality rates during outbreaks (>90%) and local extirpations (Cully and Williams 2001). As a keystone species of grassland ecosystems, prairie dog losses significantly impact numerous other species that depend on them for food or shelter, including black-footed ferrets, burrowing owls (*Athene cunicularia*), mountain plovers (*Charadrius montanus*) and several canine and avian predators (Kotliar et al. 1999). The mechanism by which *Y. pestis* is maintained in prairie and shrub-steppe ecosystems and associated species is not well understood. Some evidence suggests it circulates at enzootic levels within prairie dogs and their fleas, causing low rates of mortality (Biggins et al. 2010; Matchett et al. 2010), until reaching a threshold at which major die-offs occur (as seen in Asian plague foci, Davis et al. 2004).

Currently, plague is managed in prairie dogs through manual application of insecticides (e.g., Delta Dust^®^) to burrows to kill fleas that transmit *Y. pestis* (Biggins et al. 2010; Tripp et al. 2016). Although this method has been effective in most cases, it is labor intensive, and recent evidence from Madagascar suggests that fleas can develop resistance to the most frequently used pesticide (Boyer et al. 2014). The sylvatic plague vaccine (SPV) offers an additional approach for plague management (Abbott et al. 2012). Laboratory experiments in black-tailed and Gunnison’s prairie dogs have demonstrated that SPV provides partial protection against subcutaneous *Y. pestis* challenge (not flea bites) if delivered 6 months or more after a single consumption of vaccine-laden bait (Rocke et al. 2014, 2015). Higher levels of protection occurred after a second consumption of bait months later (Rocke et al. 2014). Vaccine safety has been demonstrated in common nontarget species (e.g., deer mice, grasshopper mice, ground squirrels), and field safety trials conducted in Colorado in 2012 found no apparent adverse effects (Tripp et al. 2015).

From 2013 to 2015, we conducted a large collaborative field study to test the effectiveness of SPV in reducing mortality from plague in four species of prairie dogs in 7 western states. This study involved state, federal, tribal and nongovernment agencies (Table 1), organized under the Black-footed Ferret Recovery Implementation Team (BFFRIT), a multiagency effort led by the US Fish and Wildlife Service. We designed the study as a matched pairs, placebo-controlled experiment. Field personnel distributing baits and collecting data were blinded to treatment assignments. Our objectives were to estimate bait uptake in prairie dogs, compare prairie dog apparent survival and relative abundance between paired SPV-treated and placebo plots, and to conduct surveillance for plague mortalities and *Y. pestis* positive fleas on our study pairs.

**Table 1:** Study Areas Included in the Phase II Sylvatic Plague Vaccine Field Trial, with the Number of Pairs at Each, Total Area (in ha), and Number of Baits Distributed Annually (2013–2015) for Colonies of Black-Tailed Prairie Dogs (BTPD), White-Tailed Prairie Dogs (WTPD), Utah Prairie Dogs (UPD), and Gunnison’s Prairie Dogs (GPD)

Study area	Lead agency	Pair designation	Species	# pairs	2013		2014		2015	
					Total area	# baits	Total area	# baits	Total area	# baits
Buffalo Gap, South Dakota	USFS	BGSD	BTPD	2	79.8	7880	85.5	10,550	88.7	10,950
Larimer County, Colorado	CPW	BTCO	BTPD	3	265.3	26,200	196.4	24,250	196.4	24,250
Charles M. Russell NWR, Montana	USFWS, USGS	CMR	BTPD	5	82.2	8406	110.2	14,055	142.6	17,538
Lower Brule, South Dakota	LBST	LBSD	BTPD	1	16.2	1600	16.2	2000	16.2	2000
Rita Blanca, Texas	USDA WS	RBTX	BTPD	2	56.7	5600	56.7	7000	56.7	7000
Wind Cave, South Dakota	NPS	WCSD	BTPD	1	16.2	1600	16.2	2000	16.2	2000
Espee Ranch, Arizona	AZGF	ERAZ	GPD	1	40.5	4000	40.5	4000	40.5	4000
Gunnison and Teller Counties, Colorado	CPW	GUCO	GPD	3	119.5	11,800	119.5	11,800	123.5	12,200
Coyote Basin, Utah	UDW	CBUT	WTPD	2	111.0	11,000	111.0	11,000	111.0	11,000
Pitchfork Ranch, Wyoming	WGF	PRWY	WTPD	2	64.8	6400	64.8	6400	64.8	6400
Cedar City, Utah	UDW	CCUT	UPD	3	29.2	3129	29.2	2885	38.1	2080
Awapa Plateau, Utah	USGS	HEUT	UPD	4	54.7	3966	54.7	5400	54.7	5400
	Total			29	936	91,581	900.7	101,340	949.3	106,485

Field sampling was conducted by the US Forest Service (USFS), Colorado Parks and Wildlife (CPW), US Fish and Wildlife Service (USFWS), US Geological Survey (USGS), Lower Brule Sioux Tribe (LBST), US Department of Agriculture Wildlife Services (USDA WS), National Park Service (NPS), Arizona Game and Fish Department (AZGF), Utah Division of Wildlife (UDW), and Wyoming Game and Fish (WGF).

## Methods

### Study Areas and Design

**Figure 1:**
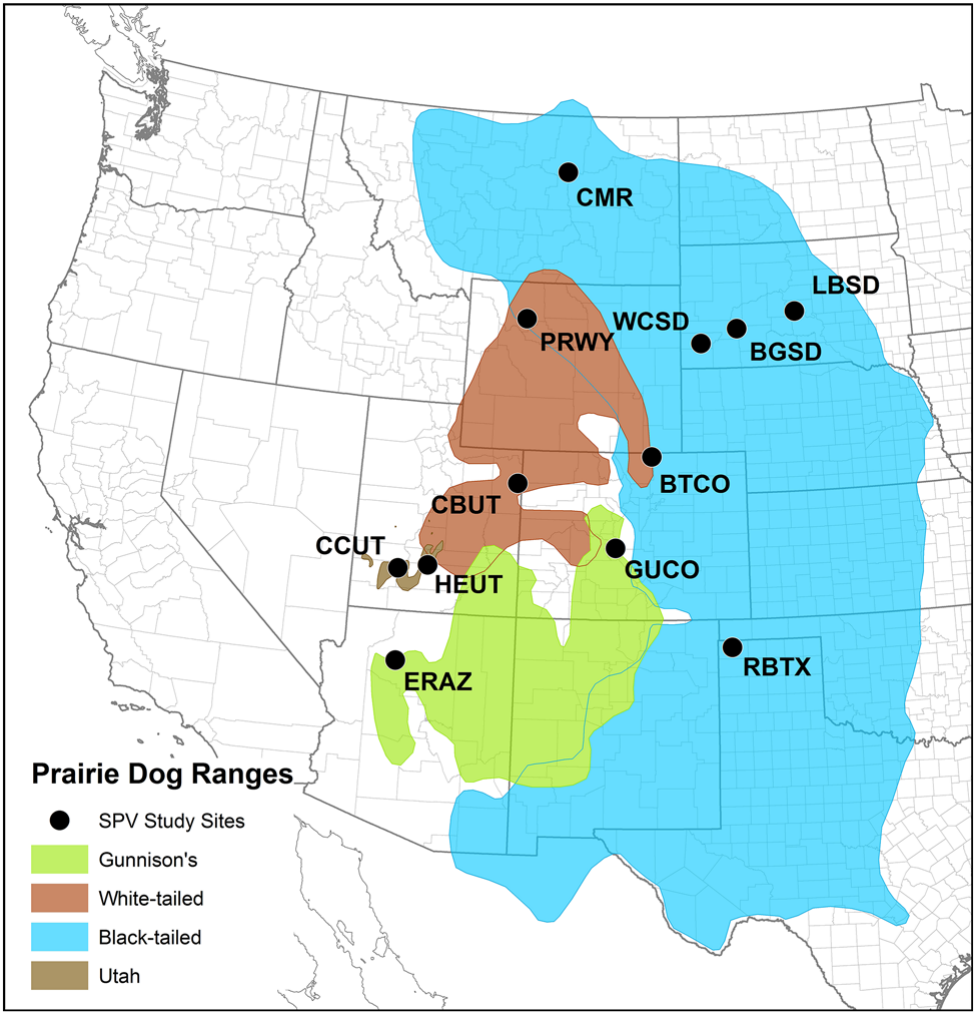
Study locations included in the SPV study by prairie dog species (*CMR* Charles M. Russell National Wildlife Refuge, MT, *LBSD* Lower Brule Sioux Tribe, SD, *WCSD* Wind Cave National Park, SD, *BGSD* Buffalo Gap National Grassland, SD, *BTCO* Larimer county, CO, *RBTX* Rita Blanca National Grassland, TX, *PRWY* Pitchfork Ranch, WY, *CBUT* Coyote Basin, UT, *ERAZ* Espee Ranch, AZ, *GUCO* Gunnison and Teller counties, CO, *CCUT* Cedar City, UT, *HEUT* high elevation (Awapa plateau), UT) (Color figure online)

Twenty-nine paired study plots (58 prairie dog colonies or plots within colonies) were selected at 12 locations in 7 states in western USA (Fig. 1; Table 1), including 14 pairs on black-tailed prairie dog colonies (*C. ludovicianus*–BTPD), 4 on Gunnison’s prairie dog colonies (*C. gunnisoni*–GPD), 4 on white-tailed prairie dog colonies (*C. leucurus*–WTPD), and 7 on Utah prairie dog colonies (*C. parvidens*–UPD). Study pairs were selected based on size (a minimum of 8 ha per plot, with a few exceptions), visual assessments of prairie dog abundance, and the availability of two plots in relatively close proximity (0.25–8 km) to serve as a pair (Figure S1). Two study pairs in Colorado (BTCO-3 and GUCO-1) had been included previously in a field safety trial in 2012 (Tripp et al. 2015), and thus 8 ha plots within these larger plots had one additional year of placebo or vaccine treatment.

### Vaccine/Bait Production and Distribution

SPV is a recombinant raccoon poxvirus (RCN-F1/V307; unlicensed Yersinia pestis Vaccine Live Raccoon Poxvirus Vector, Code 11Y2.R0) engineered to express two protective *Y. pestis* antigens, F1 and a truncated V protein (Rocke et al. 2014). SPV was reviewed and approved for experimental field use by the US Department of Agriculture Center for Veterinary Biologics. The environmental assessment and decision of record for the study are available at http://www.nwhc.usgs.gov/disease_information/sylvatic_plague. All vaccine lots used to prepare baits for this experiment were produced at the US Geological Survey National Wildlife Health Center (NWHC), as were all placebo and vaccine baits (see supplemental methods). Baits contained peanut butter as an attractant and 0.25% Rhodamine B (RB), a biomarker that is visible in hair, whiskers and feces of animals within 24 h of consumption (Fernandez and Rocke 2011; Tripp et al. 2014).

### Bait Distribution

Treatment (placebo or vaccine) was assigned to plots in a pair using a random number generator. The number of baits distributed was determined by the species and area of each pair (Table 1) and the results of previous studies (Tripp et al. 2014). Baits were distributed at a rate of 100/ha on all pairs in 2013, with the exception of HEUT pairs (averaged 51, 147, 84 and 66 baits/ha for HEUT-1, 2, 3, and 4, respectively). Due to higher densities of BTPDs than other species and in an effort to increase uptake from 2013 levels, baits were distributed at a rate of 125/ha for BTPD pairs in 2014 and 2015, while all other pairs were baited at a rate of 100/ha. The baits remained frozen until manually distributed on the colonies by walking transects and dropping them approximately every 10 m (9 m for BTPD pairs in 2014 and 2015) to ensure even distribution of baits on the colony (Tripp et al. 2014). Baits were distributed in the morning and on the same day for each member of a pair. The seasonal timing of bait distribution varied, depending on specific site considerations (weather, timing of cattle grazing, timing of hibernation, etc.), but always occurred between early June (after juveniles started to emerge from natal burrows) and early October.

### Capturing and Sampling Prairie Dogs

All animal handling and sampling procedures were approved by the NWHC Animal Care and Use Committee (Protocol#EP130214) as well as individual states as required. At least 1 week and no more than 2 months post-baiting each year, local collaborators captured, marked and sampled prairie dogs for a minimum of 3 trap days. Both plots in a pair were trapped on the same day, and, with few exceptions, trap effort (number of traps, number of days, area trapped) between plots of the same pair was similar. The goal was to capture and mark a minimum of 50 unique prairie dogs from each plot each year as previously described (Tripp et al. 2009, 2014). Sex, estimated age, and the identity of all current year and prior year recaptures were recorded for each captured animal.

For sampling all first-time captures (up to 50) each year, the animals were anesthetized with isoflurane (using vaporizers or an induction chamber) and immediately combed for fleas that were collected into tubes of sterile saline or alcohol. A small clump of hair and 3–4 whiskers were collected from each animal for assessing bait uptake through biomarker analysis. After sample collection, the animals were allowed to fully recover from anesthesia and then released at the location of capture. Identity of recaptured individuals was recorded and animals released.

Whiskers and hair were evaluated for the presence of the RB biomarker as described in Fernandez and Rocke (2011). Rates of bait uptake were calculated for each study plot and compared between adults and juveniles and placebo versus vaccine treatment for each year using an approximate test for binomial proportions (i.e., function “prop.test” in R, R Core Team 2016).

### Plague detection

Prairie dog carcasses found on or near our study pairs were collected and sent to NWHC or Colorado Parks and Wildlife (CPW) for necropsy and *Y. pestis* testing. Fleas were collected if found upon external examination. Spleen and liver tissue were sampled from all carcasses, regardless of the presumed cause of death, and tested for *Y. pestis* by standard PCR (Griffin et al. 2010) or real-time PCR (see supplemental methods). Tissues were also cultured on blood agar plates for carcasses submitted to NWHC, and suspect colonies were confirmed as *Y. pestis* using real-time PCR and probes. If *Y. pestis* was cultured from a carcass, plague was presumed as the cause of death, and it was reported as a select agent to the US Centers for Disease Control. In the absence of positive cultures, DNA was considered positive for *Y. pestis* if both the *pla* and *F1* genes amplified. Pairs with carcasses positive for *Y. pestis* by culture or PCR were classified in our analyses as “plague confirmed.”

Fleas collected from live prairie dogs and carcasses were rinsed with 70% ethanol containing 0.2% iodine, rinsed twice in sterile water, counted and identified to species (data not shown), and then pooled by species, up to 10 individuals per pool from a single animal. The pools were frozen and tested for *Y. pestis* using real-time PCR (See supplemental methods) or conventional PCR (Griffin et al. 2010). In the absence of plague-positive carcasses, if at least one flea pool from live animals on a plot tested positive by PCR, it was considered “plague suspect.”

### Prairie Dog Relative Abundance and Apparent Survival

To compare relative abundance within pairs, we used catch per unit effort (CPUE) as an index (Hopkins and Kennedy 2004), calculated as the number of unique animals captured divided by the number of trap days (Table S1). Trap days were calculated by summing the number of traps open (excluding traps that had been tripped by digging prairie dogs, nontarget captures, etc.) on each day of the trapping session. We analyzed CPUE by fitting generalized linear mixed models with “pair” as a random effect and using a logit link function for binomial data. CPUE is a binomial response (i.e., y ~ Binomial (N,p)) where y is the number of successes (unique captures), N is the number of trap days, and p is the probability of a capture. Analyses were performed in R statistical framework (R Core Team 2016) using the function “glmer” in the package “lme4” (Bates et al. 2015). We evaluated 19 different models of CPUE (Table S2), including an intercept-only model. Candidate models included the variables treatment (vaccine versus placebo), plague status (confirmed, suspect, and not detected), species, year, and treatment by year interactions as predictors. Models were evaluated using Akaike information criteria (AIC) (Burnham and Anderson 2002).

We estimated odds ratios for logistic regression models to examine effect sizes. An odds ratio for a particular covariate was calculated as e^parameter estimate^ and can be interpreted as the odds of a response = 1 given a 1 unit increase in the covariate value of interest, holding all other variables constant. Odds ratios for effects containing interaction terms were calculated by adding the appropriate parameter estimates (main effects terms + interaction effect term) prior to exponentiating. We evaluated goodness of fit by estimating Pearson’s correlation coefficient between fitted and observed CPUE.

Survival analyses were conducted using the robust design method (Kendall et al. 1995) implemented in a Bayesian framework using “rjags” in R (Plummer 2013; R Core Team 2016). The Bayesian framework provided us with greater flexibility to accommodate missing covariates and variable numbers of trapping sessions for pairs and years. To test our hypothesis that vaccine treatment had an effect on survival, we estimated parameters for models of survival containing a random effect of pair, an effect of treatment (vaccine vs placebo), plague status (plague detected vs plague not detected), age (adult vs juvenile) and interactions between plague status and treatment, and age and treatment, with four different detection functions (no covariates, plague status, treatment, and sampling effort). Plague status for survival interval t-1 to t was defined as 1 if plague was detected at the plot in year t. Once plague was detected at a plot, it was thereafter considered plague positive for the purposes of our analyses. Sampling effort was defined as the number of trapping days (range 3–10) during a season. Survival and detection estimates were logit transformed prior to estimating model coefficients. Three Markov Chain Monte Carlo (MCMC) chains were run with an adaptation phase of 10,000 iterations followed by an additional 30,000 iterations. We retained every 10th value from each chain. Models were compared using deviance information criteria (DIC) (Spiegelhalter et al. 2002); parameters were checked for convergence by visually inspecting trace plots and calculating Gelman and Rubin’s convergence diagnostic (Gelman and Rubin 1992) implemented in the R package “coda” using the function “gelman.diag.”

Fifty-two plots (26 pairs) were included in the analysis of CPUE and apparent survival. Three pairs in Utah (HEUT-1, 2, and 4) were excluded because of animal movements between adjacent plots within a pair (we documented approximately 5% of the animals moving within and between trapping sessions and between pairs, compared to <0.4% at any other pair). For one study pair (CCUT-1), only data from 2013 and 2014 were included due to complicating factors of flooding in 2015. Two pairs in Colorado (GUCO-1 and BTCO-3) were included, even though treatment began on a portion of those plots in 2012 for field safety trials (Tripp et al. 2015). Although shooting of prairie dogs was observed at CBUT-1 in 2014 and may have resulted in prairie dog declines, we retained this pair in our analyses.

## Results

Over the course of the 3-year study, 11,771 prairie dogs were captured, marked, and released (Table S1) with a total of 22,059 captures recorded from all pairs. Excluding prairie dogs that were recaptured between years, 10,249 unique animals were recorded. Samples of hair, whiskers, and fleas (if present) were collected from 6744 unique animals, some more than once if recaptured in another year. Of the 5996 animals sampled with an identifiable age (excluding HEUT-1, 2, and 4), bait uptake rates were lower for juveniles (63%, 95% C.I. 61–65) versus adults over all years (77%, 95% C.I. 76–78; *X* ^*2*^ = 159.40, *p* < 0.001). Bait uptake was similar between vaccine and placebo plots for adults and juveniles in 2013 (Table 2) but lower on vaccine plots in 2014 (adults *X* ^2^ = 5.10, *p* = 0.02; juveniles *X* ^2^ = 6.04, *p* = 0.01). In 2015, juvenile bait uptake rates were lower on vaccine plots (*X* ^2^ = 21.28, *p* < 0.001) compared to placebo plots, but adult rates were similar.

**Table 2:** Bait Uptake Rates for Placebo and Vaccine Plots for Adults and Juveniles in 2013–2015

Year	Age	% of animals that consumed bait (95% C.I)		*p* value
		Placebo	Vaccine	
2013	Adult	70 (67–74)	74 (70–77)	N.S.
	Juvenile	68 (64–72)	71 (67–75)	N.S.
2014	Adult	81 (78–85)	76 (73–79)	0.02
	Juvenile	68 (64–72)	61 (57–65)	0.01
2015	Adult	81 (77–84)	80 (77–83)	N.S.
	Juvenile	58 (53–63)	40 (35–45)	<0.001

Results of Chi-square tests for equality of proportions are reported for comparisons between placebo and vaccine plots for adults and juveniles. Comparisons that are not statistically different at *p* < 0.05 are indicated by an N.S.

### Plague Detection

Over the 3-year study, 45 prairie dog carcasses (9 BTPDs, 4 GPDs, 10 WTPDs, and 22 UPDs) were submitted for necropsy and testing to NWHC and 28 (14 BTPDs and 14 GPDs) to CPW. *Yersinia pestis* was detected in tissues from 22 carcasses at NWHC by culture and/or PCR and 10 at CPW by PCR only, providing evidence that cause of death was plague. Twenty of the plague-positive carcasses were found on vaccine plots; of those, four had consumed bait just 12–21 days prior, seven were found prior to or on the day of baiting, one was not tested, and the rest were negative for bait uptake. Only one of the 20 had been caught in years previous, and it was negative for bait uptake at that time. Other causes of prairie dog mortality were predation (5), trapping or handling mortality (16), and vehicle collision (2); cause was undetermined in 13 carcasses too decomposed for analysis. A total of 5206 and 4734 flea pools from prairie dogs were tested for *Y. pestis* DNA by PCR at NWHC and CPW, respectively, and it was found in 70 (1.3%) and 106 pools (2.2%). *Yersinia pestis* was detected in at least one prairie dog carcass or one flea pool from prairie dogs at 14 of the 29 study pairs in one or more years. In 9 cases, *Y. pestis* was detected on both members of the pair (BTCO-1, BTCO-2, BTCO-3, RBTX-1, GUCO-3, CBUT-1, CBUT-2, HEUT-1, HEUT-2). In 3 cases, *Y. pestis* was detected on the vaccine plot but not the placebo plot (ERAZ-1, GUCO-2, HEUT-3), and in 2 cases, *Y. pestis* was detected on the placebo plot but not the vaccine plot (GUCO-1, PRWY-1).

### Relative Abundance and Apparent Survival of Prairie Dogs

Although similar between plots within a pair, trapping efforts varied considerably among the 26 study pairs included in our analysis of relative abundance (Table S1). On seven study pairs, plague was confirmed as the cause of death of one or more animals, and obvious declines (>50% decrease) were noted in prairie dog relative abundance on one or both of the paired plots: BTCO-1, BTCO-2, BTCO-3, ERAZ-1, GUCO-3, CBUT-2, and HEUT-3 (Table 3). The positive plots within these 7 pairs were classified as “plague confirmed,” starting from the first year it was detected. Shortly after baiting in 2013, plague was confirmed at one study pair, BTCO-2, and complete colony collapse (>90% decline in CPUE) occurred with few animals (<1/ha) captured on either plot by 2014. Complete colony collapse also occurred on the BTCO-3 and BTCO-1 placebo plots in 2015, although the vaccine plots remained occupied. At two pairs (ERAZ-1 in 2014 and HEUT-3 in 2015), plague was confirmed on the vaccine plots, along with >50% declines in CPUE. Although plague was not detected on corresponding placebo plots, >50% declines in CPUE were noted for both in 2014.

**Table 3:**
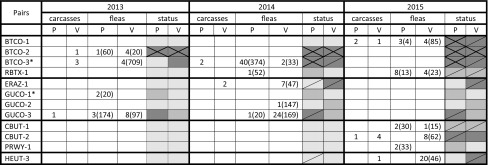
Number of Carcasses and Fleas Pools (by Number Tested) Positive for *Yersinia pestis* by Culture or PCR on the 12 Study Pairs Where Plague was Detected; *Y. pestis* was Not Detected on the Other 14 Study Pairs Included in Our Analyses (Color table online)

Treatment plots are indicated as: V-vaccine or P-placebo. *Dark orange shading* indicates plots considered “confirmed plague”; *light orange* indicates plots considered “suspect plague”; *gray* indicates plots where “plague not detected”; a *single diagonal line* indicates plots with ≥50% decline in CPUE; *crossed diagonal lines* indicate plots with ≥90% decline. Once *Y. pestis* was detected at a pair, it was thereafter considered plague positive for the purposes of our analyses.

*Pairs that were baited in 2012.

Five study pairs were classified as “plague suspect.” At these pairs, one or more *Y. pestis* positive flea pools were detected by PCR (Table 3), but no plague-positive carcasses were found. At the 14 remaining pairs, all carcasses and fleas tested negative for *Y. pestis*, and these were classified as “plague not detected.”

**Figure 2:**
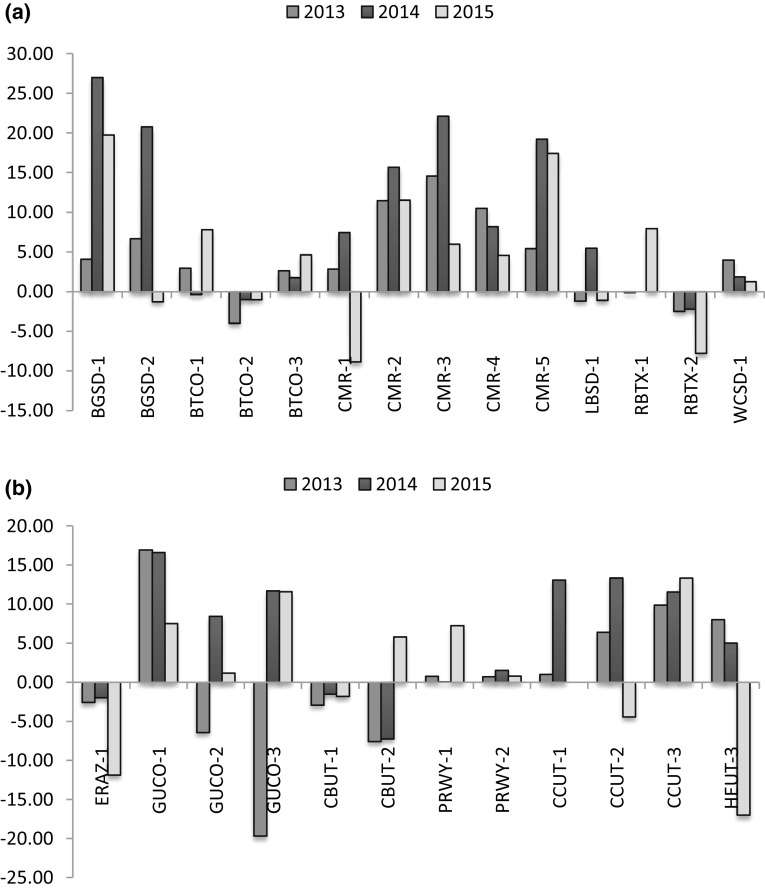
Difference in catch per unit effort (CPUE) per 100 trap days between vaccine and placebo plots by study pair for 2013–2015 for (**a**) black-tailed prairie dogs and (**b**) white-tailed, Gunnison’s and Utah prairie dogs. A positive difference is indicative of higher relative abundance on the vaccine plot compared to its matched placebo plot (Color figure online)

**Figure 3:**
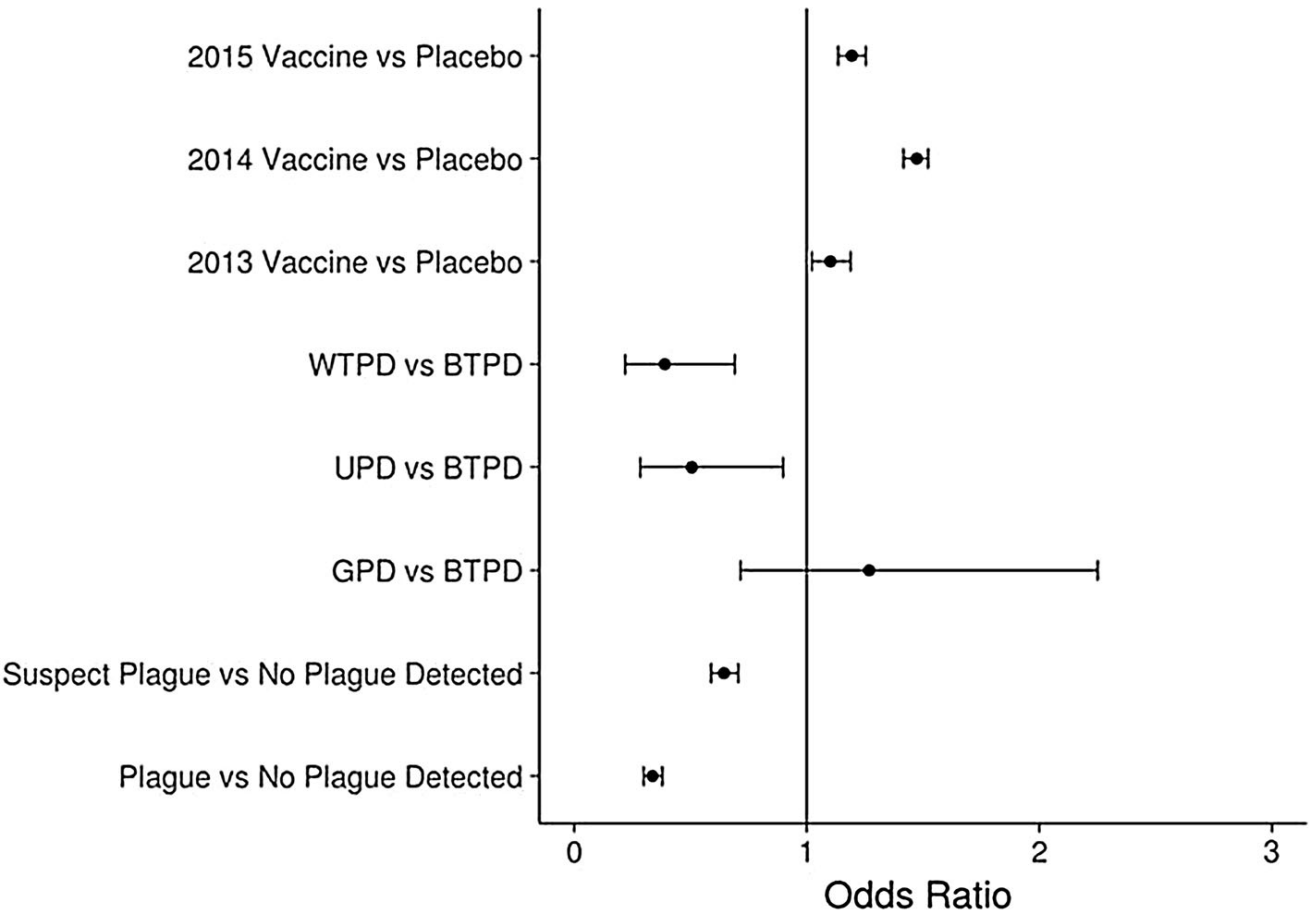
Odds ratios from best models of catch per unit effort (unique captures/trap day) as selected by AIC (Akaike Information Criteria) for 4 species of prairie dogs (*BTPD* black-tailed prairie dog, *GPD* Gunnison’s prairie dog, *WTPD* white-tailed prairie dog, and *UPD* Utah prairie dog)

Relative abundance as measured by CPUE was variable among pairs, years, and species (Fig. 2). Our best model included plague status, year, treatment, species, and treatment by year interactions and was >2 AIC points away from the intercept-only (no covariate) and the second best model (Table S2). Results indicated that vaccine treatment had an overall positive effect (*p* = 0.012) on CPUE all 3 years (Table 4, Fig. 3) that was significantly higher (*p* < 0.001) in 2014 than the other years. The odds of capture were 1.10 (95% C.I. 1.02–1.19) times higher per trap day on vaccine-treated plots than placebo plots in 2013, 1.47 (95% C.I. 1.41–1.52) times higher per trap day in 2014 and 1.19 (95% C.I. 1.13–1.25) times higher per trap day in 2015 on pairs with the same plague status (confirmed, suspect, and not detected) and the same species (Fig. 3). Removing the two pairs that started baiting in 2012 (BTCO-3 and GUCO-1) from the analysis eliminated the significant difference in CPUE in 2013 (results not shown), indicating they were responsible for the observed effect in 2013, but their removal had no effect on results for 2014 and 2015. Both confirmed and suspect plague negatively affected CPUE (*p* < 0.001). On average, odds of a capture were 0.34 (95% C.I. 0.30–0.38) and 0.64 (95% C.I. 0.59–0.71) times lower per trap day on pairs with plague confirmed and suspect, respectively, than pairs without plague detection (Table 4, Fig. 3). The model also included a species effect, indicating that CPUE was lower for WTPDs and UPDs than BTPDs and GPDs. Pearson’s correlation coefficient was 0.83 for fitted compared to observed values.

**Table 4:** Parameter Estimates from the Best Model of Catch per Unit Effort as Selected by AIC

Parameter	Estimate	Std. Error	*z* value	Pr(> |z|)
(Intercept)	−1.33	0.14	−9.47	<0.001
Plague detected versus plague not detected	−1.09	0.06	−17.94	<0.001
Suspect plague versus plague not detected	−0.44	0.05	−9.50	<0.001
2014 versus 2013	0.06	0.04	1.41	0.159
2015 versus 2013	0.44	0.04	10.81	<0.001
Vaccine versus placebo	0.10	0.04	2.50	0.012
GPD versus BTPD	0.24	0.29	0.81	0.416
UPD versus BTPD	−0.68	0.29	−2.32	<0.001
WTPD versus BTPD	−0.94	0.29	−3.23	<0.001
Vaccine*2014	0.29	0.05	5.33	<0.001
Vaccine*2015	0.08	0.05	1.47	0.142

*BTPD* black-tailed prairie dog, *GPD* Gunnison’s prairie dog, *WTPD* white-tailed prairie dog, and *UPD* Utah prairie dog.

Between year capture/recapture data included a total of 3464 animals captured in 2013, 3791 animals captured in 2014, and 3940 animals captured in 2015; 774 (22%) of the animals captured in 2013 were recaptured in 2014, and 381 (11%) were recaptured in 2015 (Table S3). Of the 3017 animals newly captured in 2014, 583 (19%) were recaptured in 2015. Two-hundred and twenty-four animals were removed from our survival analyses due to uncertain aging at first capture.

**Figure 4:**
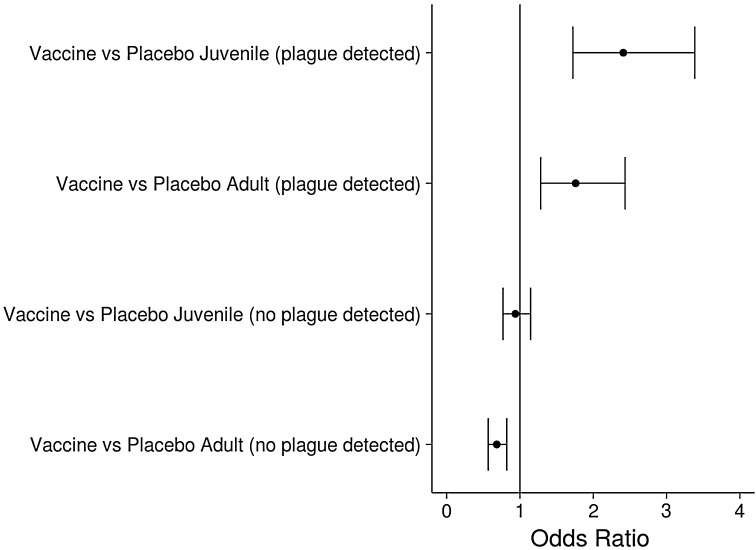
Odds ratios for comparisons of apparent survival of prairie dogs between vaccine and placebo plots on pairs with plague detected and no plague detected

The best survival model according to DIC included the effect of trapping effort on detection probability (the probability of capturing an animal if it is present; Table S4). On pairs where plague was detected, annual odds of apparent survival were 1.76 (95% B.C.I. 1.28–2.43) times higher on vaccine plots than placebo plots for adults and 2.41 (95% B.C.I. 1.72–3.33) times higher for juveniles (Fig. 4, Table S5). On pairs where plague was not detected, odds of survival were similar for juveniles between vaccine and placebo plots (0.93, B.C.I. 95% 0.77–1.45) but lower on vaccine plots for adults (0.68, B.C.I. 95% 0.57–0.82). Sampling effort was negatively associated with detection probability (i.e., pairs with more trapping days had lower detection probabilities), but this was likely an artifact of longer trapping sessions on some pairs with plague die-offs. On average, the probability of detection was higher on placebo plots (0.54; B.C.I. 95% 0.52–0.55) compared to vaccine plots (0.50; B.C.I. 95% 0.49–0.51). The odds of detection on vaccine plots were 0.95 (B.C.I. 95% 0.91–0.98) compared to placebo plots.

## Discussion

Population-level effects of vaccination can be difficult to measure, particularly in wild animals. Vaccine effectiveness depends on a combination of factors, including vaccine efficacy in individuals (measured in the laboratory) and various field conditions unique to each situation (Lahariya 2016). With regard to SPV effectiveness in prairie dogs, these factors could include species, rates of bait consumption, age and immune status of those consuming bait, area covered by baiting, and the proximity to unvaccinated populations, in addition to numerous factors related to the dynamics of *Y. pestis* infection and transmission.

Our results indicate that relative abundance (CPUE) and apparent survival were higher on SPV-treated plots during plague outbreaks, suggesting that consumption of SPV baits provided some protection for prairie dogs against plague. However, protection was incomplete on some SPV-treated plots, especially those with confirmed outbreaks, as plague-infected carcasses were detected and declines in prairie dog relative abundance were noted. It is likely that the level of vaccination within our plots (i.e., herd immunity) was insufficient to reduce the incidence of disease among unvaccinated individuals, especially in vaccine plots in close proximity to placebo plots or other adjacent untreated colonies. Even so, vaccine-treated colonies persisted in the presence of plague after just one year of baiting (e.g., ERAZ-1 and GUCO-3) and also in the face of severe die-offs, where nearby placebo plots completely collapsed (e.g., BTCO-1 and BTCO-3), including a nearby plot treated with insecticides (Tripp et al. 2017). To prevent mortality from plague, SPV must be applied proactively; without additional measures, vaccine treatment is not useful as a reactive management tool to control ongoing plague outbreaks (e.g., BTCO-2). While reactive vaccination in response to an outbreak is used for some human diseases (e.g., cholera), it is generally less effective than proactive vaccination (Azman and Lessler 2015).

Previous studies have provided evidence of enzootic maintenance of *Y. pestis* in prairie dog populations (Biggins et al. 2010; Griffin et al. 2010; Matchett et al. 2010), suggesting that *Y. pestis* transmission among prairie dogs must reach a critical threshold before noticeable die-offs occur (St. Romain et al. 2013). We detected *Y. pestis* positive fleas on live animals at one or both plots of 5 study pairs in the absence of positive carcasses, despite sampling at only one time interval per year at most pairs. We expect that low levels of *Y. pestis* transmission may have been missed or were not detectable at the other 14 study pairs, but might have been occurring at any of them. The presence of enzootic plague may explain why CPUE was higher on vaccine plots even when plague was not detected.

In our analyses of vaccine effectiveness in prairie dogs, we used measures of both apparent survival and relative abundance in pairwise comparisons between vaccine-treated and placebo plots, as neither metric alone provided a complete picture. To estimate apparent survival, we used a robust design method, but without information regarding movement of prairie dogs off trapping grids and mortality rates (few carcasses were recovered), we cannot partition apparent survival into true survival and site fidelity (Kendall et al. 1995). Prairie dog dispersal has been observed to increase after the disappearance of other coterie members (Hoogland 2013), so increased movement and emigration may be a consideration on plots where plague occurred. Recent advances in analytical methodology may allow for the estimation of dispersal and survival (see Ergon and Gardner 2014; Royle et al. 2016 for recent methods using spatial capture–recapture data); however, currently these methods are difficult to implement.

We found that apparent survival of prairie dogs was higher on vaccine-treated plots where plague was detected, but adult survival (not juveniles) was lower on vaccine plots in the absence of plague, despite findings of higher prairie dog abundance on these plots. We do not believe this finding indicates an adverse outcome of vaccine treatment, as detrimental effects have not been observed in any animals during prior laboratory and field testing (Rocke et al. 2010, 2014, 2015; Tripp et al. 2015), though additional research could be useful. Between year recapture rates were low in our study, indicating trapping effort may have been insufficient for robust survival estimates. Alternatively, a large influx of new animals (i.e., juveniles) into a population in a given year would result in increased abundance estimates without a corresponding increase in survival rates.

To evaluate population-level effects of vaccine treatment, we compared relative abundance between vaccine and placebo plots, using CPUE as an index. Although less robust than other methods, CPUE is often used when recapture rates are low (Vadell and Villafañe 2016), as they were on many of our plots. Our pairwise design controls for some of the unmeasured factors that could affect detection probability and bias our comparisons of capture rates. We found a significant positive effect of SPV treatment on CPUE, regardless of plague status, even though odds of detection on vaccine plots were slightly lower than placebo plots, indicating relative abundance may be somewhat underestimated on vaccine plots. As expected, species also had a significant effect on CPUE; UPDs and WTPDs typically occur at much lower densities than BTPDs and GPDs, and this was reflected in our results.

Although bait uptake is a critical index to vaccination, it is important to note that not all prairie dogs that consume bait respond to the vaccine or become protected against plague (Rocke et al. 2014; 2015); variables like age at vaccine consumption, number of times bait is consumed, and time between bait consumption and *Y. pestis* exposure are also important. Because seroconversion takes time and is also not always a reliable indicator of plague protection in prairie dogs (Rocke et al. 2010), for this study we prioritized assessment of bait uptake, via biomarker analysis, over serology. For the most part, bait uptake was very high in prairie dogs, over 90% on some plots, but it was significantly higher in adults than juveniles over all 3 years and plots. Bait uptake was significantly lower in juveniles on vaccine plots compared to placebo plots in 2014 and 2015, possibly due to higher relative abundance of prairie dogs found on vaccine plots. Even so, survival was higher in juveniles on vaccine plots than placebo plots in the presence of plague. Our studies have also indicated that juveniles respond better to vaccination than adults in laboratory experiments (Rocke et al. 2015), and bait uptake in the field has been shown to be higher in the fall than earlier in the year (Tripp et al. 2014). Therefore, we recommend distribution of SPV baits later in the season (August to October, depending on species) to reach juveniles that are more likely to encounter baits and survive to the next year.

In summary, we provide evidence that SPV can protect prairie dogs from plague in field settings, warranting its further evaluation as a management tool. However, vaccine treatment did not achieve full protection in this study. Some plague mortality and declines in prairie dogs were noted at several SPV-treated plots, although other mortality factors may also have played a role. In addition to timing of SPV treatment in relation to *Y. pestis* exposure, we suspect that the small size of our treatment plots and close proximity to untreated prairie dogs may influence whether the level of immunity conferred by application of vaccine-laden baits is sufficient to prevent an epizootic. We also suspect that plague protection would increase with successive years of SPV distribution as herd immunity from vaccination builds in treated populations, but these hypotheses remain to be tested. Additional fieldwork is required to optimize the use of SPV as a management tool for prairie dogs and to confirm whether its use will also provide benefits of reduced *Y. pestis* exposure to black-footed ferrets and other animals.

## Electronic supplementary material

Below is the link to the electronic supplementary material.

Supplementary material 1 (DOCX 41 kb)

## References

[CR1] Abbott RC, Osorio JE, Bunck CM, Rocke TE (2012). Forum: Sylvatic plague vaccine: a new tool for conservation of threatened and endangered species?. EcoHealth.

[CR2] Azman AS, Lessler J (2015) Reactive vaccination in the presence of disease hotspots. *Proceedings Royal Society B* 282: 20141341 (http://dx.doi.org/10.1098/rspb.2014.1341) 10.1098/rspb.2014.1341 PMC4262159 25392464

[CR3] Bates D, Maechler M, Bolker B, Walker S (2015). Fitting linear mixed-effects models using lme4. Journal of Statistical Software.

[CR5] Bhattacharya D, Bensaci M, Luker KE, Luker G, Wisdom S, Telford SR, Hu LT (2011). Development of a baited oral vaccine for use in reservoir-targeted strategies against Lyme disease. Vaccine.

[CR102] Biggins DE, Godbey JL, Gage KL, Carter LG, Montenieri JA (2010). Vector control improves survival of three species of prairie dogs (Cynomys) in areas considered enzootic for plague. Vector-borne Zoonotic Diseases.

[CR6] Boyer S, Miarinjara A, Elissa N (2014) *Xenopsylla cheopis* (Siphonaptera: Pulicidae) susceptibility to deltamethrin in Madagascar. *PLoS one* 9(11):e111998 (DOI: 10.1371/journal.pone.0111998) 10.1371/journal.pone.0111998 PMC4219825 25369291

[CR7] Burnham KP, Anderson DR (2002). Information and likelihood theory: a basis for model selection and inference. Model selection and multimodel inference: a practical information-theoretic approach.

[CR101] Cully JF, Williams ES (2001). Interspecific comparisons of sylvatic plague in prairie dogs. Journal of Mammalogy.

[CR103] Davis S, Begon M, De Bruyn L, Ageyev V, Klassovskiy N, Pole S, Viljugrein H, Stenseth N, Leirs H (2004). Predictive thresholds for plague in Kazakhstan. Science.

[CR8] Ergon T, Gardner B (2014). Separating mortality and emigration: modelling space use, dispersal and survival with robust-design spatial capture–recapture data. Methods in Ecology and Evolution.

[CR9] Freuling CM, Hampson K, Selhorst T, Schröder R, Meslin FX, Mettenleiter TC, Müller T (2013) The elimination of fox rabies from Europe: determinants of success and lessons for the future. *Philosophical Transactions of the Royal Society London B Biological Sciences* 368(1623):20120142 (DOI: 10.1098/rstb.2012.0142) 10.1098/rstb.2012.0142 PMC3720040 23798690

[CR10] Eads DA, Biggins DE (2015). Plague bacterium as a transformer species in prairie dogs and the grasslands of western North America. Conservation Biology.

[CR11] Fernandez JRR, Rocke TE (2011). The use of rhodamine B as a biomarker for oral plague vaccination of prairie dogs. Journal of Wildlife Diseases.

[CR12] Gelman A, Rubin DB (1992). Inference from iterative simulation using multiple sequences. Statistical Science.

[CR13] Griffin KA, Martin DJ, Rosen LE, Sirochman MA, Walsh DP, Wolfe LL, Miller MW (2010). Detection of *Yersinia pestis* DNA in prairie dog-associated fleas by polymerase chain reaction assay of purified DNA. Journal of Wildlife Diseases.

[CR14] Hoogland JL (2013). Prairie dogs disperse when all close kin have disappeared. Science.

[CR15] Hopkins HL, Kennedy Michael L (2004). An assessment of indices of relative and absolute abundance for monitoring populations of small mammals. Wildlife Society Bulletin.

[CR16] Kendall WL, Pollock KH, Brownie C (1995). A likelihood-based approach to capture-recapture estimation of demographic parameters under the robust design. Biometrics.

[CR17] Kotliar NB, Baker BW, Whicker AD, Plumb G (1999). A critical review of assumptions about the prairie dog as a keystone species. Environmental Management.

[CR18] Lahariya C (2016). Vaccine epidemiology: a review. Journal of Family Medicine and Primary Care.

[CR20] Matchett MR, Biggins D, Kopsco V, Powell B, Rocke TE (2010). Enzootic plague reduces black-footed ferret (*Mustela nigripes*) survival in Montana. Vector-borne and Zoonotic Diseases.

[CR21] Murphy D, Costello E, Aldwell FE, Lesellier S, Chambers MA, Fitzsimons T, Corner LA, Gormley E (2014). Oral vaccination of badgers (*Meles meles*) against tuberculosis: comparison of the protection generated by BCG vaccine strains Pasteur and Danish. The Veterinary Journal.

[CR23] Plummer M (2013) rjags: Bayesian graphical models using MCMC. R package version 3-10. https://CRAN.R-project.org/package=rjags

[CR25] R Core Team (2016) R: a language and environment for statistical computing, Vienna, Austria: R Foundation for Statistical Computing. URL https://www.R-project.org/.

[CR26] Rocke TE, Pussini N, Smith S, Williamson J, Powell B, Osorio JE (2010). Consumption of baits containing raccoon pox-based plague vaccines protects black-tailed prairie dogs (*Cynomys ludovicianus*). Vector-borne and Zoonotic Diseases.

[CR27] Rocke TE, Kingstad-Bakke B, Berlier W, Osorio JE (2014). A recombinant raccoon poxvirus vaccine expressing both *Yersinia pestis* F1 and truncated V antigens protects animals against lethal plague. Vaccines.

[CR28] Rocke TE, Tripp DW, Lorenzsonn F, Falendysz E, Smith S, Williamson J, Abbott R (2015). Age at vaccination may influence response to sylvatic plague vaccine (SPV) in Gunnison’s prairie dogs (*Cynomys gunnisoni*). EcoHealth.

[CR100] Rossi S, Staubach C, Blome S, Guberti V, Thulke H-H, Vos A, Koenen F, Le Potier M-F (2015). Controlling of CSFV in European wild boar using oral vaccination: a review. light orange indicates. Frontiers in Microbiology.

[CR29] Royle JA, Fuller AK, Sutherland C (2016). Spatial capture–recapture models allowing Markovian transience or dispersal. Population Ecology.

[CR31] Slate D, Algeo TP, Nelson KM, Chipman RB, Donovan D, Blanton JD, Niezgoda M, Rupprecht CE (2009). Oral rabies vaccination in North America: opportunities, complexities, and challenges. PLoS Neglected Tropical Diseases 22.

[CR32] Spiegelhalter DJ, Best NG, Carlin BP, Van Der Linde A (2002). Bayesian measures of model complexity and fit. Journal of the Royal Statistical Society: Series B (Statistical Methodology).

[CR33] St. Romain K, Tripp DW, Salkeld DJ, Antolin MF (2013). Duration of plague (*Yersinia pestis*) outbreaks in black-tailed prairie dog (*Cynomys ludovicianus*) colonies of northern Colorado. Ecohealth.

[CR34] Tripp DW, Gage KL, Montenieri JA, Antolin MF (2009). Flea abundance on black-tailed prairie dogs (*Cynomys ludovicianus*) increases during plague epizootics. Vector-Borne and Zoonotic Diseases.

[CR35] Tripp DW, Rocke TE, Streich SP, Brown NL, Ramos J, Miller MW (2014). Season and application rates affect vaccine bait consumption by prairie dogs. Journal of Wildlife Diseases.

[CR36] Tripp DW, Rocke TE, Streich SP, Abbott RC, Osorio JE, Miller MW (2015) Apparent field safety of a raccoon poxvirus-vectored plague vaccine in free-ranging prairie dogs, Colorado, USA. *Journal of Wildlife Diseases* (DOI: 10.7589/2014-02-051) 10.7589/2014-02-051 25588006

[CR37] Tripp DW, Streich SP, Sack DA, Martin DJ, Griffin KA, Miller MW (2016). Season of deltamethrin application affects flea and plague control in white-tailed prairie dog colonies. Journal of Wildlife Diseases.

[CR38] Tripp DW, Rocke TE, Runge JP, Abbott RC, Miller MW (2017). Burrow dusting or oral vaccination prevents plague-associated prairie dog colony collapse. EcoHealth.

[CR39] Vadell MV, Villafañe IEG (2016). Environmental variables associated with hantavirus reservoirs and other small rodent species in two national parks in the Paraná Delta, Argentina: implications for disease prevention. Ecohealth.

