# Supplementary material for: Sylvatic Plague Vaccine Partially Protects Prairie Dogs (Cynomys spp.) in Field Trials
Source: Ecohealth. 2017 Jun 22;14(3):438–50. doi: 10.1007/s10393-017-1253-x (PMC5662665; doi:10.1007/s10393-017-1253-x)
Supplement: Supplementary file 1 — Supplementary material 1 (DOCX 41 kb) [file 10393_2017_1253_MOESM1_ESM.docx]

**SUPPLEMENTAL MATERIAL**

**Supplemental Methods**

**Vaccine production**

All SPV lots used for the experiment were produced at the USGS National Wildlife Health Center (NWHC) by inoculation of Vero cell monolayers cultured in 1264 cm^2^ cell factories (Nunc; Cat#140250).  After complete infection of cells within approximately 48 hours of incubation at 37°C with 5% C0_2,_the cell factories were frozen horizontally at -80°C.  After thawing, the suspension was centrifuged 15,317 RCF for 20 min at 4°C.  The resulting pellets were dislodged with 10mM TRIS, sonicated on ice, and vortexed, after which they were aliquoted in 1ml and 30ml volumes and frozen at -80°C.  A 1ml aliquot of each lot of virus was then titrated on Vero cells 3 times to determine average number of plaque forming units (pfu) per ml and stored back for quality control testing.  Periodically, these stored lots were tested for bacterial and mycoplasma contamination by culture, as well as specific viruses by PCR.

**Bait Production**

All placebo and vaccine baits were produced at NWHC. Baits were made using a powdered formula (FoodSource Lures; Birmingham, AL) previously shown to be palatable for prairie dogs (Fernandez and Rocke 2011). The bait powder was mixed with water at 70°C using an industrial mixer. Organic peanut butter (17% w/w) was added as an attractant, as well as 0.25% Rhodamine B (RB), a biomarker that is visible in hair, whiskers and feces of animals within 24 hours of consumption (Fernandez and Rocke 2011). After this mixture was cooled to 40°C, the vaccine virus was added in a quantity equivalent to a dosage of 5x10^7^ pfu/bait after thorough mixing. The bait mixture was then spread onto 9 ½” x 14 ¾” trays and allowed to solidify at room temperature. Once solid, the baits were cut into square pieces weighing approximately 4.5g and then placed in a walk-in incubator (20°C) and dried for 4 days. Placebo baits were made similarly, but no vaccine virus was added. After the drying period, the baits were vacuum-sealed, labeled, and frozen at -20°C. A small number of baits from each lot produced were stored frozen for quality control purposes and retained after field distribution. These were periodically checked for vaccine virus titer.

***Y. pestis* Detection in Carcasses**

DNA was extracted from approximately 20mg of tissue using the Wizard SV Genomic DNA Purification System (Promega; Madison, WI).  Prior to PCR amplification, the DNA samples were diluted to a final concentration of 50ng/µl. PCR was performed using the DNA Engine Opticon System (BioRad; Hercules, CA) and results were analyzed using the Opticon Monitor Version 3.1 software. Real-time PCR primers and probes were designed using the RealTime® PCR Tool software (Integrated DNA Technology; Coralville, IA), the caf1 gene sequence located on the *Y. pestis* pG8786 plasmid (Genbank accession NC_006323) and the pla gene located on the *Y. pestis* pPCP1 plasmid (Genbank accession AL109969). ***F1* gene**: forward primer - 5’cagccaggatttctttgttcg 3’; reverse primer - 5’cggttacggttacagcatcag 3’; probe sequence - 5’tgcaagtttaccgcctttggaacc 3’. Amplification was performed using a profile of 50°C for 2 min, 95°C for 10 min, 40 cycles of 95°C for 15 sec and 62°C for 30 sec, followed by a final incubation at 45°C for 5 min. ***Pla* gene**: forward primer - 5’ attggacttgcaggccagtatc 3’; reverse primer – 5’ ataacgtgagccggatgtcttc 3’. Amplification was performed using SYBR Green PCR Master Mix (Life Technologies; Carlsbad, CA), 500nM of each primer and a profile of 50°C for 2 min, 95°C for 10 min, 40 cycles of 95°C for 15 sec and 58°C for 1 min, followed by a final incubation at 45°C for 5 min. In all amplification runs a positive control (*Yersinia pestis-*CO92; BEI Resources) and a no template control (NTC) reactions were included.

If *Y. pestis*was cultured from a carcass, plague was presumed as the cause of death, and it was reported as a select agent to the Centers for Disease Control.  In the absence of positive cultures, DNA was only considered positive for *Y. pestis* if both the pla and F1 genes were present.

***Y. pestis* Detection in Fleas**

At NWHC, flea DNA was prepared using a combination of bead-beating, proteinase K treatment, and DNA extraction.  Fleas were homogenized for 30 seconds at a setting of 8 using a Bullet Blender Storm 24 bead beater and a 3:2 combination of 2.0mm and 1.0mm zioxide beads, respectively (Next Advance; Averill Park, NY). Following the brief beating, PBS was added to the samples to bring the total volume to 100µl. Fifty microliters was transferred to a new 1.5ml Safe-Lock tube (Eppendorf; Hauppauge, NY) for proteinase K treatment (50µl of a 100µg prot K solution in 2x digestion buffer [Zymo Research; Irvine, CA] and an overnight incubation at 55°C) while the remaining 50µl of sample was stored back for future analyses. Following proteinase K treatment, DNA extractions were performed using the Zymo Quick gDNA Miniprep Kit (>1 flea) or Micro Kit (1 flea) (Zymo Research; Irvine, CA) depending on the number of fleas in each pool. DNA samples were then screened for the *pim* gene, which resides on the pPCP1 plasmid of *Y. pestis*, using real-time PCR. *Pim* gene**:** forward primer - 5’ ttgtctcatggcatctatggg 3’; reverse primer - 5’ tccatgagttgtctttattgataagtg 3’; probe sequence - 5’ actcggtttgcttgaaggttgtctgt 3’. Amplification was performed using the following profile: 50°C for 2 min, 95°C for 10 min, 40 cycles of 95°C for 15 sec and 58°C for 30 sec, followed by a final incubation at 45°C for 5 min. Reactions included 50ng sample DNA, 500nM of each primer, 200nM probe and the TaqMan Universal PCR Master Mix (Life Technologies; Carlsbad, CA). Samples with a Ct value of ≤34.0 were considered as suspect positive. Suspect positives were then confirmed by testing for the *F1* gene as described above; a Ct value of ≤34.0 was considered positive.  The rest of the samples were archived for future analyses.

**Supplemental Results**

**Table S1.** Treatment, number of unique animals captured (N), number of trap days, and size of trap area (ha) by study plot and year. NT=not trapped.

| Study plot | Treatment | 2013 | | | | 2014 | | | | 2015 | | | |
| --- | --- | --- | --- | --- | --- | --- | --- | --- | --- | --- | --- | --- | --- |
|  |  | N | trap days | trap area (ha) | % bait uptake | N | trap days | trap area (ha) | % bait uptake | N | trap days | trap area (ha) | % bait uptake |
| BGSD-1A | vaccine | 56 | 255 | 4.92 | 52 | 131 | 214 | 4.82 | 38 | 78 | 210 | 4.36 | 57 |
| BGSD-1B | placebo | 59 | 330 | 3.98 | 47 | 81 | 237 | 4.38 | 73 | 59 | 339 | 3.55 | 62 |
| BGSD-2A | placebo | 55 | 330 | 4.36 | 44 | 81 | 248 | 5.06 | 32 | 52 | 190 | 4.53 | 60 |
| BGSD-2B | vaccine | 56 | 240 | 4.84 | 64 | 126 | 234 | 4.68 | 44 | 61 | 230 | 4.40 | 57 |
| BTCO-1A | vaccine | 60 | 483 | 17.24 | 96 | 91 | 388 | 13.17 | 88 | 47 | 499 | 16.56 | 98 |
| BTCO-1B | placebo | 46 | 485 | 16.95 | 85 | 91 | 382 | 14.98 | 84 | 0 | 500 | 15.81 | NS |
| BTCO-2A | vaccine | 11 | 697 | 12.95 | 58 | 3 | 199 | 8.05 | 0 | 7 | 250 | 6.45 | 33 |
| BTCO-2B | placebo | 43 | 665 | 13.73 | 63 | 6 | 198 | 7.87 | 33 | 11 | 246 | 4.92 | 73 |
| BTCO-3A | placebo | 130 | 566 | 20.45 | 75 | 4 | 270 | 8.51 | 0 | 3 | 386 | 12.81 | 33 |
| BTCO-3B | vaccine | 147 | 577 | 20.30 | 93 | 55 | 1449 | 30.86 | 92 | 35 | 646 | 19.07 | 91 |
| CBUT-1A | vaccine | 17 | 388 | 9.93 | 89 | 50 | 272 | 10.88 | 94 | 8 | 351 | 12.77 | 63 |
| CBUT-1B | placebo | 29 | 396 | 10.39 | 89 | 59 | 297 | 14.38 | 84 | 21 | 487 | 16.19 | 70 |
| CBUT-2A | vaccine | 12 | 270 | 10.10 | 83 | 43 | 387 | 12.25 | 69 | 57 | 551 | 13.05 | 36 |
| CBUT-2B | placebo | 50 | 520 | 8.99 | 76 | 53 | 287 | 11.26 | 86 | 8 | 176 | 5.70 | 43 |
| CCUT-1A | vaccine | 33 | 225 | 4.14 | 88 | 44 | 228 | 3.65 | 77 | NT | NT | NT | NT |
| CCUT-1B | placebo | 35 | 222 | 4.53 | 91 | 14 | 225 | 2.58 | 86 | NT | NT | NT | NT |
| CCUT-2A | placebo | 18 | 219 | 4.41 | 78 | 20 | 225 | 2.88 | 95 | 40 | 225 | 5.22 | 90 |
| CCUT-2B | vaccine | 32 | 225 | 3.59 | 63 | 50 | 225 | 3.21 | 78 | 31 | 225 | 4.65 | 77 |
| CCUT-3A | vaccine | 47 | 225 | 2.37 | 45 | 39 | 225 | 1.61 | 59 | 67 | 225 | 3.14 | 56 |
| CCUT-3B | placebo | 12 | 189 | 2.19 | 58 | 13 | 225 | 1.34 | 31 | 37 | 225 | 2.03 | 54 |
| CMR-1A | placebo | 41 | 332 | 8.44 | 58 | 59 | 194 | 8.67 | 87 | 61 | 176 | 13.68 | 84 |
| CMR-1B | vaccine | 53 | 342 | 8.80 | 76 | 78 | 206 | 8.30 | 83 | 49 | 190 | 13.15 | 78 |
| CMR-2A | placebo | 45 | 228 | 6.84 | 64 | 70 | 222 | 8.68 | 65 | 102 | 190 | 10.95 | 78 |
| CMR-2B | vaccine | 67 | 212 | 8.18 | 70 | 93 | 197 | 10.02 | 79 | 120 | 184 | 11.52 | 77 |
| CMR-3A | vaccine | 84 | 220 | 8.01 | 54 | 109 | 202 | 8.58 | 71 | 100 | 199 | 11.20 | 75 |
| CMR-3B | placebo | 51 | 216 | 8.67 | 82 | 72 | 226 | 9.61 | 82 | 89 | 201 | 10.46 | 92 |
| CMR-4A | placebo | 50 | 217 | 7.98 | 60 | 74 | 213 | 8.05 | 83 | 59 | 204 | 11.20 | 84 |
| CMR-4B | vaccine | 73 | 213 | 6.14 | 66 | 88 | 205 | 8.71 | 84 | 77 | 176 | 11.41 | 92 |
| CMR-5A | vaccine | 60 | 190 | 8.36 | 58 | 107 | 201 | 9.80 | 67 | 97 | 146 | 12.80 | 71 |
| CMR-5B | placebo | 49 | 188 | 7.64 | 84 | 65 | 191 | 7.93 | 85 | 72 | 149 | 9.28 | 91 |
| ERAZ-1A | vaccine | 48 | 402 | 7.13 | 94 | 20 | 642 | 13.87 | 95 | 18 | 449 | 8.09 | 69 |
| ERAZ-1B | placebo | 59 | 399 | 4.90 | 82 | 32 | 627 | 7.56 | 77 | 69 | 434 | 5.55 | 84 |
| GUCO-1A | vaccine | 159 | 389 | 10.79 | 86 | 178 | 379 | 10.69 | 81 | 155 | 386 | 10.49 | 75 |
| GUCO-1B | placebo | 96 | 401 | 13.22 | 80 | 119 | 392 | 11.99 | 94 | 128 | 392 | 11.05 | 69 |
| GUCO-2A | vaccine | 157 | 399 | 11.74 | 23 | 180 | 388 | 11.44 | 33 | 108 | 397 | 10.37 | 42 |
| GUCO-2B | placebo | 180 | 391 | 14.05 | 33 | 148 | 390 | 13.30 | 79 | 103 | 392 | 13.36 | 82 |
| GUCO-3A | vaccine | 123 | 394 | 9.59 | 61 | 82 | 389 | 10.64 | 66 | 81 | 387 | 9.80 | 47 |
| GUCO-3B | placebo | 199 | 391 | 9.69 | 54 | 37 | 394 | 7.45 | 69 | 37 | 396 | 6.70 | 38 |
| HEUT-1A | placebo | 71 | 504 | 5.84 | 15 | 61 | 623 | 7.74 | 90 | 20 | 498 | 7.69 | 79 |
| HEUT-1B | vaccine | 49 | 477 | 3.59 | 22 | 32 | 619 | 7.00 | 96 | 22 | 484 | 6.71 | 80 |
| HEUT-2A | placebo | 83 | 560 | 2.92 | 47 | 106 | 712 | 3.38 | 56 | 23 | 347 | 3.59 | 86 |
| HEUT-2B | vaccine | 44 | 560 | 2.69 | 43 | 84 | 730 | 4.42 | 53 | 19 | 360 | 3.17 | 83 |
| HEUT-3A | placebo | 41 | 375 | 5.12 | 68 | 18 | 506 | 6.80 | 94 | 66 | 281 | 8.28 | 69 |
| HEUT-3B | vaccine | 68 | 350 | 4.67 | 84 | 80 | 449 | 7.42 | 74 | 20 | 298 | 5.05 | 58 |
| HEUT-4A | placebo | 30 | 504 | 3.32 | 93 | 24 | 264 | 3.64 | 96 | 57 | 286 | 5.01 | 94 |
| HEUT-4B | vaccine | 38 | 630 | 3.59 | 91 | 14 | 242 | 5.00 | 100 | 44 | 268 | 5.26 | 88 |
| LBSD-1A | vaccine | 64 | 1513 | 7.22 | 92 | 177 | 968 | 7.65 | 67 | 166 | 972 | 7.65 | 58 |
| LBSD-1B | placebo | 81 | 1446 | 7.22 | 86 | 128 | 999 | 7.65 | 76 | 158 | 868 | 7.65 | 68 |
| PRWY-1A | vaccine | 69 | 742 | 18.20 | 94 | 120 | 1264 | 17.86 | 85 | 369 | 1153 | 17.85 | 83 |
| PRWY-1B | placebo | 65 | 762 | 16.66 | 100 | 118 | 1249 | 17.07 | 93 | 301 | 1216 | 17.08 | 76 |
| PRWY-2A | placebo | 57 | 1553 | 17.48 | 96 | 96 | 1262 | 17.33 | 94 | 195 | 1186 | 17.32 | 82 |
| PRWY-2B | vaccine | 65 | 1486 | 16.38 | 95 | 115 | 1261 | 17.41 | 81 | 204 | 1184 | 17.41 | 83 |
| RBTX-1A | vaccine | 50 | 117 | 6.58 | 50 | 29 | 89 | 4.69 | 41 | 14 | 89 | 4.48 | 85 |
| RBTX-1B | placebo | 51 | 119 | 5.68 | 42 | 30 | 89 | 4.73 | 31 | 6 | 90 | 5.02 | 17 |
| RBTX-2A | vaccine | 40 | 120 | 6.23 | 80 | 18 | 90 | 4.67 | 94 | 23 | 90 | 4.62 | 26 |
| RBTX-2B | placebo | 43 | 120 | 6.26 | 44 | 20 | 90 | 4.75 | 95 | 31 | 90 | 4.31 | 53 |
| WCSD-1A | placebo | 79 | 353 | 6.10 | 68 | 55 | 390 | 6.00 | 35 | 73 | 379 | 6.03 | 56 |
| WCSD-1B | vaccine | 84 | 315 | 6.17 | 65 | 57 | 363 | 6.93 | 62 | 52 | 252 | 4.90 | 64 |

**Table S2.** Candidate models for catch per unit effort (all models include a random effect for pair).

| **Candidate models** | **df** | **AIC** | **∆AIC** |
| --- | --- | --- | --- |
| plague status, year, treatment, species, treatment*year | 12 | 3523.36 | 0.00 |
| plague status, year, treatment, treatment*year | 9 | 3530.23 | 6.87 |
| plague status, year, treatment, species | 10 | 3549.91 | 26.55 |
| plague status, year, treatment | 7 | 3556.74 | 33.38 |
| plague status, year, species | 9 | 3648.95 | 125.59 |
| plague status, year | 6 | 3655.69 | 132.33 |
| plague status, species, treatment | 8 | 3818.72 | 295.36 |
| plague status, treatment | 5 | 3823.56 | 300.20 |
| year, treatment, species, treatment*year | 10 | 3876.93 | 353.57 |
| year, treatment, species | 8 | 3898.34 | 374.98 |
| year, treatment | 5 | 3898.53 | 375.17 |
| year, species | 7 | 3922.66 | 399.30 |
| year | 4 | 3927.50 | 404.14 |
| plague status, species | 7 | 4002.40 | 479.04 |
| plague status | 4 | 4002.64 | 479.28 |
| treatment, species | 6 | 4026.01 | 502.65 |
| treatment | 3 | 4026.03 | 502.67 |
| species | 5 | 4132.32 | 608.96 |
| intercept only model | 2 | 4132.39 | 609.03 |

**Table S3.** Number of prairie dogs captured and recaptured on placebo and vaccine plots.

| **Plot treatment** | **Year** | **First captured** | **Recaptured 2014** | **Recaptured 2015** |
| --- | --- | --- | --- | --- |
| Placebo | 2013 | 1686 | 345 (20%) | 189 (11%) |
|  | 2014 | 1227 | NA | 234 (19%) |
| Vaccine | 2013 | 1778 | 429 (24.0%) | 192 (11%) |
|  | 2014 | 1790 | NA | 349 (19%) |

**Table S4.** Model selection results for different detection functions for survival models containing the effects of vaccination, plague status, plague status*treatment, age, and age*treatment.

| **Effects on detection** | **DIC** |
| --- | --- |
| sampling effort | 65426 |
| plague status | 66357 |
| treatment | 66393 |
| no covariates | 66400 |

**Table S5.** Parameter estimates from best model of Bayesian analyses of survival as selected by DIC. The median value, upper (UCI) and lower (LCI) 95% Bayesian credible intervals are presented.

| **Parameter** | **Median** | **LCI** | **UCI** |
| --- | --- | --- | --- |
| Detection intercept | 0.148 | 0.099 | 0.197 |
| Detection effort | -0.131 | -0.140 | -0.122 |
| Survival intercept | -1.514 | -1.963 | -1.089 |
| Survival vaccine vs placebo | -0.063 | -0.262 | 0.136 |
| Survival plague detected vs no plague detected | -0.740 | -1.016 | -0.463 |
| Survival plague detected*vaccine | 0.945 | 0.582 | 1.316 |
| Survival adult vs juvenile | 0.776 | 0.587 | 0.964 |
| Survival adult*vaccine | -0.314 | -0.565 | -0.067 |

A. B.

**Figure S1.** A. Initial size in hectares of study pairs by plot and species. B. Distance between matched pairs in kilometers, measured from the center of each trapping area. Black-tailed prairie dog pairs are blue, Gunnison’s prairie dog pairs are green, white-tailed prairie dog pairs are brown, and Utah prairie dog pairs are orange.
